# Sleep quality, sleep hygiene practices and their influencing factors among Malaysian university students: A cross-sectional study

**DOI:** 10.51866/oa.717

**Published:** 2025-04-13

**Authors:** Shree Vijayan Lakshme, Raja Sharranesh, Yeoh Yong Khang, Harbaksh Singh Arvinder-Singh

**Affiliations:** 1 BSc Biomedical Sciences, Faculty Of Medicine, Asian Institute of Medicine, Science and Technology (AIMST), Bedong, Kedah, Malaysia. Email: lakshreeshabu@gmail.com; 2 BSc Biomedical Sciences, Faculty Of Medicine, Asian Institute of Medicine, Science and Technology (AIMST), Bedong, Kedah, Malaysia.; 3 BSc Biomedical Sciences, Faculty Of Medicine, Asian Institute of Medicine, Science and Technology (AIMST), Bedong, Kedah, Malaysia.; 4 MBBS, MSc Health Research, PhD, Community Health (Epid and Stats) Clinical Research Centre, Hospital Raja Permaisuri Bainun Ipoh, Ministry of Health Malaysia.; 5 Faculty Research Support Unit (FRSU), Faculty of Medicine & Health Sciences, UPM, Malaysia.

**Keywords:** Sleep quality, Sleep hygiene, Students, Sleep disorders

## Abstract

**Introduction:**

Sleep quality refers to the subjective experience of sleep, encompassing aspects such as duration, depth and continuity. In contrast, sleep hygiene practices involve behaviours and habits conducive to healthy sleep patterns. This study aimed to explore the prevalence of good sleep quality and hygiene and the factors that affect good sleep hygiene and quality.

**Methods:**

A cross-sectional study was conducted among 384 Malaysian university students sampled from October to December 2023 using a validated self-administered questionnaire that included the Pittsburgh Sleep Quality Index (PSQI) and Sleep Hygiene Index (SHI). The PSQI and SHI were used to measure sleep quality and hygiene, respectively. Data was analysed using SPSS v26.

**Results:**

Approximately 94.0% (95% Confidence Interval (CI)=91.0-96.1) of the participants had good/normal sleep hygiene, while 60.2% (95% CI=55.1-65.1) had poor sleep quality. The multivariate binary logistic regression analysis showed that the participants who had good sleep hygiene had a 4.36-fold (95% CI=1.26–15.17, P=0.02) higher odds of having good sleep quality. Conversely, ethnicity (high odds ratio, P<0.001) and sleep hygiene were associated with a 4.22-fold (95% CI=1.19–14.95, P=0.03) higher odds of good sleep quality.

**Conclusion:**

Malaysian university students have a high prevalence of good sleep hygiene, but many have poor sleep quality. Although sleep hygiene may be directly affected by sleep quality, sleep quality can be affected by sleep hygiene and ethnicity.

## Introduction

Sleep quality is defined as an individual’s self-satisfaction with all aspects of the sleep experience.^1^ It encompasses several dimensions, including sleep latency (how quickly one falls asleep), duration (total time spent asleep), continuity (ability to stay asleep throughout the night) and depth (perceived restfulness and rejuvenation upon waking).^1^ A person’s health and welfare depend on the quality of their sleep.^1^

Sleep hygiene refers to a set of behavioural and environmental guidelines aimed at fostering restful sleep.^2^ Patients are taught healthy sleeping practices during sleep hygiene education.^2^ Initially developed to address mild-to-moderate insomnia, sleep hygiene education includes a list of suggestions (e.g. avoid coffee, exercise frequently, remove noise from the sleeping area and have a regular sleep schedule) to enhance the quality of sleep.^2^

Both sleep hygiene and sleep quality are critical for overall health and well-being.^3^ It is important to address these issues because a previous study found that 70.6% of 313 undergraduate students at a public university in Malaysia had poor sleep quality, indicating that a large number experienced daytime sleepiness.^3^ Sleep quality was also linked to excessive daytime sleepiness and increased stress levels.^3^ Some scholars have proposed a bi-directional relationship between sleep hygiene and sleep quality, where sleep hygiene impacts sleep quality, and poor sleep quality can deteriorate sleep hygiene over time. While sleep hygiene is traditionally seen as a predictor of sleep quality, the relationship may be bi-directional.^2^ Emphasising that the mechanism is still not fully understood could set the stage for further exploration.

For students, sleep quality and sleep hygiene are important, as they affect their cognitive function.^4^ Both sleep hygiene and sleep quality are highly associated with each other and can potentially affect cognitive function.^4^ Poor sleep can negatively impact memory, attention and decision-making abilities.^4^ Research has indicated that sleep significantly impacts memory function in both humans and animals.

Insomnia, or the inability to fall and stay asleep, affects about 30% of the population and can lead to increased emotional distress, social difficulties and reduced physical health.^5^ Consistent sleep disruption can result in higher rates of accidents, absenteeism, decreased job performance, lower quality of life and greater healthcare utilisation.^5^ Sleep quality has also been linked to non-communicable diseases such as hypertension, diabetes and other metabolic diseases.^6^ A Malaysian study found that sociodemographic factors including age, sex and examination performance did not significantly affect students’ sleep quality, despite the common belief that older adults experience less sleep quality and more night-time awakenings.^7^

Many factors affect both sleep quality and sleep hygiene. Environmental factors such as noise, light and temperature can significantly impact sleep quality.^5^ Lifestyle choices such as caffeine and alcohol consumption, smoking, irregular sleep schedules and a lack of physical activity can disrupt sleep.^2^ Physiological factors, including stress, anxiety and depression, are major contributors to sleep disturbances.^7^ Excessive use of electronic devices, especially before bedtime, can interfere with sleep patterns by disrupting circadian rhythms.^4^ Although socio-demographics might not significantly affect sleep quality, other research has highlighted that younger students, particularly those in demanding academic environments, often experience poor sleep quality, although cognitive behavioural therapy for insomnia has shown effectiveness in improving sleep hygiene, enhancing sleep quality and reducing insomnia symptoms.^6^

Despite widespread knowledge about the importance of good sleep practices, many individuals, particularly students, struggle to implement these habits effectively. According to a study conducted in Malaysia, students are well aware of and knowledgeable about good sleep hygiene and quality.^8^ However, the results demonstrated how poorly Universiti Tun Hussein Onn Malaysia students practised good sleep hygiene and quality habits.^8^ Nonetheless, good knowledge and awareness do not correspond with good sleep hygiene and quality habits.^8^ This shows the need to increase awareness of sleep quality and hygiene practices among students, as it will assist students in adjusting their sleep practices, helping them cope with stress and attain better academic achievements.

This study aimed to explore sleep quality, sleep hygiene practices and their influencing factors among Malaysian university students. By understanding these elements, the study sought to identify potential interventions to improve sleep health in this population.

## Methods

### Study design

This cross-sectional study was conducted in Malaysia from October to December 2023 among university students. Participants were chosen from both public and private local universities. We utilised social media platforms to disseminate questionnaires to potential participants. Participants were asked for online informed consent before proceeding (Google Forms link: https://forms. gle/ggdrh7f15ezkWzm59). After agreeing to participate, participants were asked a series of demographic questions before answering the questionnaire identifying their sleep quality and sleep hygiene habits. The responses to the questionnaire were kept strictly anonymous and were available only in English.

### Study sample

The sample size was determined using OpenEpi version 3 (SS Propor module), an open-source calculator. To determine the appropriate sample size, we calculated the sample sizes separately for sleep quality (70.6% prevalence) and sleep hygiene (50% assumed prevalence). The required sample sizes were 323 and 384, respectively. Thus, we used 384 as the minimum sample size to ensure sufficient power for both sleep hygiene and sleep quality. The population size was set to an infinite population, the confidence limit to 95% and the design effect to 1. We included students who were conveniently sampled based on the receipt of the questionnaire link. Students who were studying overseas and students aged below 17 years were excluded from this study.

### Data collection tools

We utilised two validated tools to collect data to answer our research questions and general objectives. We utilised the Pittsburgh Sleep Quality Index (PSQI) and Sleep Hygiene Index (SHI)^9,10^ to measure sleep quality and hygiene, respectively. Although no pilot study was conducted, the tools used in this research have been utilised in previous studies on similar populations in Malaysia, with reliability testing conducted.^11^

The SHI is a 13-item self-administered index for assessing the presence of behaviours thought to compromise sleep hygiene. Each item is scored from 0 to 4, indicating the responses of ‘never’, ‘rarely’, ‘sometimes’, ‘frequently’ or ‘always’, respectively. The scores are then added to obtain the global SHI, where higher scores indicate maladaptive sleep hygiene status.^10^ They are re-categorised into a binary outcome of having either poor sleep hygiene (score of 35-52) or normal/good sleep hygiene (score of 34 and below). The items constructing the SHI were derived from the diagnostic criteria for inadequate sleep hygiene in the International Classification of Sleep Disorders.^12^ The SHI has been shown to correlate positively with features of inadequate sleep hygiene (P<0.01) and all components of the PSQI (P<0.05).^10^

The PSQI is a validated questionnaire with a total of seven components: subjective sleep quality, sleep latency, sleep duration, habitual sleep efficiency, sleep disturbances, use of sleeping medications and daytime dysfunctions. These components contain 19 self-rated questions, each scored from 0 to 3. A score of 3 indicates the greatest disturbance, while a score of 0 shows the least disturbance. The scores are then added to obtain the global PSQI, where higher scores indicate poorer sleep quality.^9^ They are categorised into a binary outcome of having either poor sleep quality (score of 6-21) or normal/good sleep quality (score of 0-5). The PSQI has been demonstrated to have good internal reliability, stability over time and evidence of validity and is well regarded in the sleep research community.

Data collection was conducted using Google Forms, through which participants completed the SHI and PSQI self-administered questionnaires. Information about participants and the usage of their data was clearly explained before their enrolment into the study.

### Data analysis

The chi-square test was used to analyse the relationship between the independent categorical variables (sex, ethnicity, living arrangement, field of study and campus location) and categorical outcomes of the SHI and PSQI. Multivariate binary logistic regression was used to predict the SHI and PSQI based on the independent variables. A goodness-of-fit model test was conducted for the final model, using the Nagelkerke R^2^ value, Hosmer-Lemeshow test and percentage of correctly classified cases. A Nagelkerke R^2^ value of 0.7 or more was preferred, along with a correctly classified percentage of ≥70% and a Hosmer-Lemeshow P-value of >0.05.

Variables that yielded a P-value of ≤0.3 in the univariate analysis were included in the final model for the multivariate analysis.^13^ A P-value of <0.05 was deemed significant for the multivariate model. All univariate odds were reported as odds ratios (ORs) and multivariate odds as adjusted ORs. The chi-square test was conducted to determine significant differences between the categories of the SHI and PSQI and Pearson’s correlation analysis (normally distributed data) to evaluate the magnitude and strength of the correlation between the SHI and PSQI. An *r* value of ≥0.7 indicated a good correlation when the P-value was <0.05.^13,14^ An r value of 0.5-0.7 indicated a moderate correlation.

## Results

The demographic details of the participants were compared against the outcomes of sleep hygiene and sleep quality. Sleep hygiene was assessed using the SHI and sleep quality using the PSQI.

### Sleep hygiene

**Prevalence of sleep hygiene**

Among the 384 participants, 361 (94.0%, 95% Confidence Interval (CI)=91.0–96.1) had good/ normal sleep hygiene, while 23 (6.0%, 95% CI=3.9-9.0) had poor sleep hygiene.

**Univariate analysis**

The univariate analysis was conducted via the chi-square test (Table 1). The demographic variables as well as the PSQI were compared against the SHI. Ethnicity (P=0.22) and sleep quality (P=0.01) were found to yield a P-value of ≤0.3 and were thus included in the multivariate analysis. A detailed comparison of the sleep hygiene outcome with the demographic variables is shown in Table 1.

**Table 1:** Comparison of the demographic factors with sleep hygiene based on the Sleep Hygiene Index.

Variable	Poor sleep hygiene N_1_=23 n (%)	Normal/good sleep hygiene N_2_=361 n (%)	P-value
Sex^*^			
Male	10 (6.4)	147 (93.6)	0.89
Female	13 (5.9)	208 (94.1)	
Non-binary	0	3 (100)	
Ethnicity^**^			
Chinese	5 (3.3)	148 (96.7)	
Indian	12 (7.2)	155 (92.8)	**0.22**
Malay	6 (10.2)	53 (89.8)	
Others	0	3 (100)	
Living arrangement			
Alone	2 (11.8)	15 (88.2)	
Off-campus hostel	3 (3.4)	85 (96.6)	0.69
On-campus hostel	12 (6.6)	171 (93.4)	
Weekday at hostel/weekend with family	0	1 (100)	
With family	6 (6.3)	89 (93.7)	
Field of study^***^			
Arts and humanities	3 (7.3)	38 (92.7)	
Business and eco	5(7.5)	62 (92.5)	
Engineering	5 (11.4)	39 (88.6)	0 56
Foundation	0	5(100)	
Health and medicine	6 (3.8)	153 (96.2)	
Law	0	7 (100)	
Science and technology	4 (6.7)	56 (93.3)	
Campus location			
Johor	1 (10.0)	9 (90.0)	
Kedah	7 (6.1)	108 (93.9)	
Kelantan	0	2 (100)	
Kuala Lumpur	8 (7.8)	94 (92.2)	
Melaka	1 (16.7)	5 (83.3)	
Negeri Sembilan	0	2(100)	0 87
Pahang	1 (5.0)	19 (95.0)	
Penang	0	24 (100)	
Perak	3(6.1)	46 (93.9)	
Perlis	1 (14.3)	6(85.7)	
Selangor	0	26 (100)	
Terengganu	1 (5.9)	16 (94.1)	
East Malaysia	0	4 (100)	
Sleep quality			
(Pittsburgh Sleep Quality Index)			0 01
Poor	20 (8.7)	211 (91.3)	
Good	3 (2.0)	150 (98.0)	

* 3 missing

** 2 missing

*** 1 missing

In 2x2 situations where the expected count was <5, Fisher’s exact test was applied. In situations where a >2x2 table was obtained and an expected count of <5 was seen, an exact test was applied.

**Regression analysis**

The multivariate binary logistic regression analysis was conducted to compare the factors that affect the sleep hygiene outcome and determine potential confounders (Table 2).

**Table 2:** Univariate and multivariate analyses comparing significant demographic factors with good sleep hygiene.

Variable	OR (95% CI)	P-value	AOR (95% CI)	P-value
Ethnicity				
Indian Malay Others Chinese	0.44 (0.15 to 1.27) 0.30 (0.09 to 1.0.19) >1000 (<0.001 to >1000) Reference	**0.22**	0.57 (0.2 to 1.67) 0.35 (0.10 to 1.22) >1000 (<0.001 to >1000) Reference	0.44
Sleep quality (Pittsburgh Sleep Quality Index)				
Good Poor	4.74 (1.38 to 16.23) Reference	**0.01**	4.36 (1.26 to 15.17) Reference	**0.02**

The regression analysis compared good/normal sleep hygiene against poor sleep hygiene based on the SHI.

**Multivariate binary logistic regression**

Ethnicity and sleep quality were found to yield a P-value of ≤0.3. After being analysed in the multivariate analysis, good sleep quality was associated with a 4.36-fold (95% CI=1.26-15.17, P=0.02) higher odds of good sleep hygiene compared to poor sleep quality. Ethnicity was found to be a confounder. Full details are listed in Table 2.

**Goodness of fit**

The goodness-of-fit model test was conducted for the final model (multivariate model). The Nagelkerke R2 value was calculated at 0.084; the Hosmer-Lemeshow test yielded a P-value of 0.72; and the correctly classified percentage was 94%. The model fit was deemed to be adequate and good.

### Sleep Quality

**Prevalence of sleep quality**

Among the 384 participants, 231 (60.2%, 95% CI=55.1–65.1) had poor sleep quality, whereas 153 (39.8%, 95% CI=34.9-44.9) had good sleep quality.

**Univariate analysis**

The univariate analysis was first conducted via the chi-square test. From the univariate analysis, it was found that ethnicity (P<0.001), living arrangements (P=0.01) and sleep hygiene (P<0.001) significantly differed between good and poor sleep quality based on the PSQI. These factors were then included in the multivariate logistic regression analysis. Full details are shown in Table 3 below.

**Table 3:** Comparison of the demographic factors and sleep quality based on the Pittsburgh Sleep Quality Index.

Variable	Poor sleep quality N_1_=231 n (%)	Good sleep quality N_2_=153 n (%)	P-value
Sex^*^			
Male	94 (59.9)	63 (40.1)	0.97
Female	133 (60.2)	88 (39.8)	
Non-binary	2 (66.7)	1 (33.3)	
Ethnicity^**^			
Chinese Indian Malay Others	72 (47.1) 117 (70.1) 37 (62.7) 3 (100)	81 (52.9) 50 (29.9) 22 (37.3) 0	**<0.001**
Living arrangement			
Alone Off-campus hostel On-campus hostel Weekday at hostel/weekend with family With family	13 (76.5) 42 (47.7) 121 (66.1) 0 55 (57.9)	4 (23.5) 46 (52.3) 62 (33.9) 1 (100) 40 (42.1)	0.01
Field of study^***^			
Arts and humanities Business and eco Engineering Foundation Health and medicine Law Science and technology	28 (68.3) 39 (58.2) 29 (65.9) 4 (80.0) 92 (57.9) 6 (85.7) 32 (53.3)	13 (31.7) 28 (41.8) 15 (34.1) 1 (20.0) 67 (42.1) 1 (14.3) 28 (46.7)	0.41
Campus location			
Johor Kedah Kelantan Kuala Lumpur Melaka Negeri Sembilan Pahang Penang Perak Perlis Selangor Terengganu East Malaysia	9 (90.0) 72 (62.6) 2 (100) 53 (52.0) 5 (83.3) 2 (100) 15 (75.0) 14 (58.3) 27 (55.1) 4 (57.1) 13 (50.0) 12 (70.6) 3 (75.0)	1 (10.0) 43 (37.4) 0 49 (48.0) 1 (16.7) 0 5 (25.0) 10 (47.1) 22 (44.9) 3 (42.7) 13 (50.0) 5 (29.4) 1 (25.0)	0.48
Sleep quality (Pittsburgh Sleep Quality Index)			
Poor Good	211 (91.3) 20 (87.0)	150 (8.7) 3 (13.0)	<0.001

* 3 missing

** 2 missing

*** 1 missing

In 2x2 situations where the expected count was <5, Fisher’s exact test was applied. In situations where a >2x2 table was obtained and an expected count of <5 was seen, an exact test was applied.

**Regression analysis**

The regression analysis compared good sleep quality against poor sleep quality based on the PSQI.

**Multivariate binary logistic regression**

Ethnicity, living arrangements and sleep hygiene were found to yield a P-value of ≤0.3. After being analysed in the multivariate analysis, ethnicity (large OR with P<0.001 because a cell had a 0 value reported) and normal/good sleep hygiene were associated with a 4.22-fold (95% CI=1.19–14.95, P=0.03) higher odds of good sleep quality compared to poor sleep hygiene. Living arrangements were found to be a confounder. Although ethnicity was deemed to be significant from the large OR obtained, caution is needed when considering it a factor affecting good sleep quality. Full details are presented in Table 4.

**Table 4:** Univariate and multivariate analyses comparing the significant demographic factors with good sleep quality.

Variable	OR (95% CI)	P-value	AOR (95% CI)	P-value
Ethnicity				
Indian Malay Others Chinese	>1000 (<0.001 to >1000) >1000 (<0.001 to >1000) >1000 (<0.001 to >1000) Reference	**<0.001**	>1000 (<0.001 to >1000) >1000 (<0.001 to >1000) >1000 (<0.001 to >1000) Reference	**<0.001**
Living arrangement				
Off-campus hostel/apartment On-campus hostel Weekday at hostel/weekend with family With family Alone	3.56 (1.08 to 11.77) 1.67 (0.52 to 5.32) >1000(<0.001 to >1000) 2.36 (0.72 to 7.79) Reference	**0.02**	2.60 (0.74 to 9.05) 1.26 (0.37 to 4.23) >1000 (<0.001 to >1000 2.21 (0.64 to 7.65) Reference	0.06
Sleep Hygiene Index (SHI)				
Good Poor	4.74 (1.38 to 16.23) Reference	**0.01**	4.22 (1.19 to 14.95) Reference	0.03

**Goodness of fit**

The goodness-of-fit model test was conducted for the final model. The Nagelkerke R^2^ value was calculated at 0.131; the Hosmer-Lemeshow test yielded a P-value of 0.79; and the correctly classified percentage was 63.1%. The model fit was deemed to be adequate and moderate.

### Comparison between sleep quality and sleep hygiene

Table 5 shows the chi-square analysis comparing the PSQI and SHI. Among the 231 participants who had poor sleep quality, 91.3% had normal/good sleep hygiene. Among the 153 participants who had good sleep quality, 98% had normal/good sleep hygiene. The P-value yielded was 0.01. Thus, there was a significant difference between sleep quality and sleep hygiene. From the eyeball method, sleep quality could be considered rather poor compared to sleep hygiene, causing a significant difference. Pearson’s correlation analysis was conducted between the SHI and PSQI, yielding a P-value of <0.001. However, the correlation coefficient was 0.45, which indicated that the correlation between the two was positive but was rather poor.

**Table 5:** Chi-square analysis of the relationship between the PSQI and SHI.

	SHI binary		Total	P-value
	Poor N_1_=23 n (%)	Normal/good N_2_=361 n (%)		
PSQI				
Poor sleep quality	20 (8.7)	211 (91.3)	231 (100)	**0.01**
Good sleep quality	3 (2.0)	150 (98.0)	153(100)	

PSQI: Pittsburgh Sleep Quality Index, SHI: Sleep Hygiene Index

## Discussion

This study revealed an intricate link between sleep hygiene and sleep quality among Malaysian university students, corresponding to reports across various age groups and regions indicating strong associations between these factors.^14-17^ Factors such as psychological problems, stress, exposure to tobacco smoke, pain, family issues, patience, air quality, intense physical activity, noise, room scents, depression, anxiety and tension have been identified as common causes of poor sleep experiences.^18^

In this study, the majority of the students (60.2%) reported poor sleep quality, which is consistent with the findings of a previous study on Pakistani medical students, where 40% reported poor sleep quality, with 72% going to bed after midnight.^19^ Similarly, another study showed a global PSQI of 6.9±3.2, with the majority of participants (62.7%) being classified as poor sleepers.^20^ Students also experienced poor sleep in studies conducted in Iran, China, Hong Kong and Qatar, which involved a combination of students from various faculties.^16,21-23^ Although the majority of the students in the present study were medical students, the trends among other faculty students might be similar due to shared stressors such as academic pressure. However, in a study sampling students from Lebanon and Ethiopia, it was reported that they were classified as having good sleep, highlighting potential cultural and lifestyle influences that might have caused differences in the findings.^24,25^

Some studies have reported a strong correlation between the female sex and poor sleep quality, with 60.2% of female students experiencing poor sleep, possibly due to physiological needs and changes (e.g. hormonal fluctuations associated with the menstrual cycle, pregnancy and menopause) or increased frequency of sleep disorders (e.g. restless leg syndrome) and disturbances.^19,20,23^

In another study, a significant proportion of the Malaysian university student population experienced insomnia and poor sleep quality due to unhealthy habits such as smoking, drinking and compensatory sleep.^26^ Addressing these habits is essential to improve sleep quality.^26^ Strategies that might help include quitting smoking and alcohol consumption, promoting consistent sleep schedules and focusing on subjective sleep quality.^26,27^ This being consistent with the results of our study might suggest that poor sleep quality could be due to regular tobacco and alcohol consumption.^16,22^ Therefore, subjective sleep quality might be the answer to identifying good and poor sleeping practices.

Previous research has also reported a link between delayed sleep phase syndrome (DSPS), which is characterised by reduced sleep during the week and regular sleep on weekends, and poor sleep quality among students.^4,28^ Among the factors influencing it is financial independence, as it may introduce additional responsibilities, such as balancing work and academics, leading to irregular sleep patterns, stress and the risk of developing DSPS.^4,28^

The current study revealed discrepancies in sleep hygiene among the students, with most (>90%) reporting good/normal sleep hygiene. This contradicts the findings in an Iranian study where male students scored worse on four measures of sleep hygiene than female students, although female students performed worse in two areas.^23^

In this study, both science and medical students experienced poor sleep quality, which might be due to factors that were reported in another study, including balancing work, study and training hours.^20^ This differs from the findings of a study conducted in Qatar, where medical students had the best sleep quality (mean PSQI of 6.7), which was attributed to their use of better strategies for managing academic obligations, greater knowledge and practice of sleep hygiene and differences in their study curriculum.^21^

About 13% of the participants in our study reported having poor sleep hygiene but good sleep quality. This mirrors previous findings where a smaller proportion of respondents (30.8%) exhibited high sleep quality despite poor sleep hygiene.^15^ These findings suggest that individuals can experience good sleep quality despite poor sleep hygiene.^15^

According to the literature, individuals who are aware of good sleep hygiene practices tend to have regular sleep patterns and higher sleep quality.^17^ Conversely, those with low awareness of good sleep practices are more likely to develop poor sleep behaviours.^17^ This might imply that education and awareness about good sleep hygiene are potentially critical factors in improving sleep quality among students. However, knowledge alone is insufficient, as it must be coupled with consistent practice of good sleep habits.

In a previous study, it was reported that while most students had no significant sleep difficulties (e.g. trouble falling asleep, difficulty breathing, waking up in the middle of the night, experiencing pain or having nightmares), poor sleep hygiene could lead to suboptimal sleep quality.^29^ On average, students reported getting 8.5 hours of sleep per day, which aligns with the recommendations of the National Sleep Foundation for adolescents. However, factors such as bedtime procrastination and inconsistent sleep schedules could compromise the quality of sleep - something that could have been a factor for the student population sampled in the current study.^29^

Research has shown that poor sleep quality and hygiene might significantly affect the academic performance and general well-being of students and might be linked to non-communicable diseases.^5-7,22^ Sleep hygiene education and awareness can improve sleep patterns, leading to better mental health and academic outcomes.^5,7,22^ Conversely, neglecting these aspects can result in chronic sleep issues, negatively impacting students’ physical health, cognitive function and emotional stability.^5,7,22^ A cross-sectional study conducted among 500 medical students at the International Islamic University Malaysia, Kuantan, showed that students generally had poor sleep quality (59.4%).^30^ Poor sleep quality was independently reported among students with depression symptoms especially those in their pre-clinical years.^30^

### Strengths and limitations

A key strength of this study is its detailed examination of sleep quality and hygiene, addressing a gap in the Malaysian university student context. However, despite providing valuable insights, this study has limitations. The reliance on self-reported data may introduce bias, and the cross-sectional design limits causal inferences. Additionally, the sample, predominantly medical students with limited ethnic diversity, may not represent the actual composition of Malaysian university students.

### Future research

Future research should explore tailored sleep interventions, with implications for policy, such as implementing university programmes to promote better sleep habits and addressing the unique challenges students face while balancing work and study. Future research should also utilise longitudinal designs with stratified sampling according to ethnicity to evaluate the possibility of an association with sleep quality.

## Conclusion

Approximately 60.2% of Malaysian university students experience poor sleep quality. Sleep quality is affected by sleep hygiene and ethnicity, while sleep hygiene depends on sleep quality. Although there is a significant difference in the correlation of the PSQI and SHI that yielded a positive directional association, the correlation is poor. This suggests a need for targeted interventions to address specific sleep behaviours, including procrastination, smoking, alcohol drinking and sex differences, which are perhaps unique challenges faced by university students.

### Recommendations

As many non-communicable diseases are now linked to sleep medicine, general practitioners (doctors who come into contact with the largest number of patients) should consider screening university students on their sleep quality based on sleep hygiene and ethnicity.

Based on the study findings, good sleep hygiene does not necessarily lead to good sleep quality, as there are other possible influencing factors. Hence, it is important to consider other factors such as addiction (smoking, drinking and drug use), mental health status and obesity-related factors in future research to determine whether there is an association with sleep hygiene/quality. All these are common primary care screening questions that should be practised to better enhance holistic medicine among adolescents and young adults.

## Data sharing statement

Raw data from this study will not be provided via controlled access repositories to maintain participant confidentiality. Upon a reasonable request, the corresponding author will provide the data supporting the study's conclusions.

Researchers who fulfil the requirements for access to confidential information will be granted access to the data, which includes responses to the questionnaire used in the investigation.

**How does this paper make a difference in general practice?**

- General practitioners can use the findings by integrating sleep quality assessments into routine check-ups and offering resources or referrals for sleep improvement for university students.
- Sleep hygiene, sleep quality and their influencing factors (e.g. mental health challenges) should be screened to assist general practitioners in assessing sleep issues holistically.
- The findings can also be used by general practitioners working with student populations in educational institutions to screen for sleep issues, promote awareness and offer resources to support consistent sleep patterns and optimise sleep environments.
- Screening adolescents and young adults thoroughly for sleep habits is important for the future control of non-communicable diseases in the country, as sleep medicine has been linked to future comorbidities.
