# Corrigendum to “Sleep Quality, Sleep Hygiene Practices and Influencing Factors Among Malaysian University Students”

**DOI:** 10.51866/cor.003

**Published:** 2026-03-30

**Authors:** Shree Vijayan Lakshme, Raja Sharranesh, Yeoh Yong Khang, Harbaksh Singh Arvinder-Singh

**Affiliations:** 1 Faculty Of Medicine, Asian Institute of Medicine, Science and Technology (AIMST), Bedong, Kedah, Malaysia.; 2 Community Health (Epid and Stats) Clinical Research Centre, Hospital Raja Permaisuri Bainun Ipoh, Ministry of Health Malaysia.; 3 Faculty Research Support Unit (FRSU), Faculty of Medicine & Health Sciences, UPM, Malaysia.

We, the authors of the article titled *“Sleep quality, sleep hygiene practices and their influencing factors among Malaysian university students: A cross-sectional study'* published in Malaysian Family Physician,^1^ would like to bring to the reader’s attention the minor corrections identified in the published version of our paper.

We, the authors, received feedback highlighting certain typological errors in the aforementioned article. We acknowledge that these corrections are typographical and reference-related in nature and do not affect the results, interpretations, or conclusions of the study. We kindly request that the following amendments be made to the online version of the article.

In page 3, 2nd paragraph of the article, the line “ They are categorised into a binary outcome of having either poor sleep quality (score of 0–5) or normal/good sleep quality (score of 6–27). The sentence should be corrected to read as “They are categorised into a binary outcome of having either poor sleep quality (score of 6–21) or normal/good sleep quality (score of 0–5).”.^2^ The scoring was an overlooked typographical error. On page 3 of the article, under the section describing statistical analysis, the sentence “An r value of >0.7 indicated a good correlation when the P-value was <0.05.” currently cites Reference 13 only. An additional reference should be included to support the statistical interpretation. Therefore, Reference 14 should be added immediately after Reference 13.

The newly added reference will read as follows:

14. numiqo Team (2025). numiqo: Online Statistics Calculator. numiqo e.U., Graz, Austria. Available at: https://numiqo.com

As a result of this addition, all subsequent references should be renumbered accordingly throughout the manuscript to maintain sequential citation order. Similarly, the last row of Table 4 mentions “Pittsburgh Sleep Quality Index” should be corrected to “Sleep Hygiene Index (SHI).” The last correction would be of an incomplete reference on page 10, “Arvinder-Singh HS. Comparison of the COVID-19 mortality occurring in hospitals and those brought in dead within Malaysia. PubMed. 2022;77(3):338–346.” The reference should be corrected to read as “ Arvinder-Singh HS. Comparison of the COVID-19 mortality occurring in hospitals and those brought in dead within Malaysia. *Med JMalaysia.* 2022;77(3):338–46.”

There were minor typological errors in the document that may have affected the assistance of understanding the scientific value, and some of the methodology and interpretation of the results. Thus, we have now submitted this document to clarify that so that it might help the readers understand better what was elucidated in the document. We apologise to the readers for these oversights and appreciate the opportunity to rectify them. We confirm that all other information presented in the article remains accurate.
